# Auditory function and brainstem responses in allergic rhinitis: systematic review and meta-analysis

**DOI:** 10.1590/2317-1782/e20240307en

**Published:** 2025-10-13

**Authors:** Thales Rafael Correia de Melo Lima, Brenda Carla Lima Araújo, Silvia de Magalhães Simões, Paulo Ricardo Martins-Filho

**Affiliations:** 1 Programa de Pós-graduação em Ciências da Saúde, Hospital Universitário, Universidade Federal de Sergipe – UFS - Aracaju (SE), Brasil.; 2 Departamento de Fonoaudiologia, Universidade Federal de Sergipe – UFS - São Cristóvão (SE), Brasil.; 3 Serviço de Alergia e Imunologia, Departamento de Medicina, Hospital Universitário, Universidade Federal de Sergipe – UFS - Aracaju (SE), Brasil.

**Keywords:** Rhinitis, Hearing Loss, Audiometry, Evoked Response, Systematic Review, Meta-Analysis

## Abstract

**Purpose:**

To investigate differences in auditory thresholds and brainstem evoked response audiometry (BERA) between individuals with and without allergic rhinitis (AR).

**Research strategies:**

Searches were conducted in PubMed, Scopus, Web of Science, Google Scholar, and Open Access Theses and Dissertations on April 2, 2024. The study protocol was registered on the Open Science Framework (https://doi.org/10.17605/OSF.IO/XUTSN).

**Selection criteria:**

Observational studies comparing auditory outcomes between individuals with and without AR were included.

**Data analysis:**

The Joanna Briggs Institute checklist was used to assess bias. The primary outcome was the mean difference (MD) in auditory thresholds (250Hz to 8000Hz) measured by audiometry. Secondary outcomes included latencies of waves and interpeak intervals. The meta-analysis was conducted using the inverse variance method under a random effects model.

**Results:**

Five studies with 432 participants were included. Meta-analysis revealed higher auditory thresholds in patients with AR at 4000Hz (MD = 7.83 dB; 95% CI: 2.46 to 13.19; p = 0.004) and 8000Hz (MD = 8.66 dB; 95% CI: 2.70 to 14.62; p = 0.004). No differences were observed for frequencies <4000Hz or in BERA outcomes.

**Conclusion:**

This meta-analysis identified significantly higher auditory thresholds at 4000Hz and 8000Hz in individuals with AR, suggesting a potential peripheral auditory effect. No consistent differences were found in BERA parameters. These findings suggest that AR may impact high-frequency hearing without affecting neural conduction at the brainstem level.

## INTRODUCTION

Allergic rhinitis is often characterized by symptoms such as nasal congestion, rhinorrhea, post-nasal drip, sneezing, and itching in the eyes, nose, and throat, and is commonly associated with the presence of other conditions including asthma, eczema, chronic or recurrent sinusitis, cough, tension headaches, and migraines^(1)^. Hearing loss represents a partial or total decline in the ability to hear, emerging as a multifactorial condition with etiologies ranging from noise exposure to infections, aging, traumatic events, and health comorbidities^(2)^.

The World Health Organization identifies hearing loss as responsible for more than 5% of the global prevalence of disabilities, positioning it as the fourth leading cause of disability worldwide^(3)^. The repercussions of hearing loss transcend the reduction in hearing capacity, affecting language comprehension, sound localization, mental health, social inclusion and participation, and significantly impacting labor productivity^(4,5)^. In the pediatric context, the repercussions extend to impairments in linguistic development, academic performance, and socioemotional evolution, highlighting the importance of early detection and effective interventions^(6-8)^.

Recent scientific investigations suggest a potential connection between allergic rhinitis and the incidence of hearing loss^(9,10)^. The central hypothesis is that the inflammation of the nasal mucosa characteristic of allergic rhinitis may extend to the surrounding tissues, obstructing the drainage ostium of the auditory tube, which would adversely affect its functionality and induce negative pressure in the middle ear. This pressure change can compromise the mobility of the ossicular chain and the integrity of the tympanic membrane, resulting in auditory alterations^(11,12)^.

Studies have shown that patients with allergic rhinitis exhibit higher rates of middle ear dysfunction, including Eustachian tube dysfunction and serous otitis media, leading to conductive hearing loss^(9)^. A systematic review by Cheng et al.^(13)^ showed that individuals with allergic rhinitis have a higher risk of otitis media with effusion (OME), a condition that can cause temporary or even permanent hearing impairment. Furthermore, recent studies suggest that allergic inflammation may contribute to auditory dysfunction through Eustachian tube involvement, potentially affecting middle ear function and sound transmission, even in the absence of overt middle ear pathology^(14)^. These findings emphasize the need for further research to elucidate the mechanisms linking allergic rhinitis to auditory dysfunction and reinforce the clinical importance of evaluating hearing in patients with persistent rhinitis symptoms.

Despite the preliminary bodies of evidence, the literature lacks systematic reviews and meta-analyses that consolidate and synthesize the available evidence on the relationship between allergic rhinitis and auditory alterations. Therefore, the purpose of this systematic review and meta-analysis was to investigate potential differences in auditory thresholds and brainstem auditory evoked potentials between individuals diagnosed with allergic rhinitis compared to individuals without rhinitis.

## METHODS

This systematic review and meta-analysis followed the Preferred Reporting Items for Systematic Reviews and Meta-Analyses (PRISMA)^(15)^ statement (Supplementary file). The protocol for this study was registered on the Open Science Framework (https://doi.org/10.17605/OSF.IO/XUTSN).

### Research question and eligibility criteria

This study focused on the following question: Are there differences in auditory thresholds and brainstem auditory evoked potentials between individuals with allergic rhinitis compared to those without such condition? Studies were considered eligible if they met the following criteria:

- Type of study: observational.
- Population of interest: individuals diagnosed with rhinitis, regardless of age and gender.
- Comparison: individuals without rhinitis.
- Outcomes of interest: auditory threshold and brainstem auditory evoked potentials.

In this review, editorials, comments, opinions, reflective articles, protocols, technical reports, reviews, and articles addressing other conditions not related to rhinitis and hearing loss were excluded. Studies that did not provide sufficient raw data to assess the relationship between rhinitis and hearing loss were also excluded, such as auditory threshold values, the number of individuals with hearing loss among those with rhinitis, diagnostic criteria for hearing loss, and the number of patients with rhinitis.

### Search strategy

The search for studies was conducted in the PubMed, Scopus, and Web of Science databases. A grey literature search was conducted using Google Scholar (first 100 results) and Open Access Theses and Dissertations (OATD). The search was limited to studies published in full versions up to April 2, 2024, without language restrictions. The reference lists of all eligible studies and reviews were also assessed to identify additional studies for inclusion.

The search strategy was first developed for PubMed using the descriptors “Rhinitis”, “Hearing Loss”, “Hearing Disorders”, and “Deafness”, along with their synonyms. Boolean operators AND and OR were used to combine concepts and synonyms, respectively. The strategy was then adapted for other databases, considering differences in indexing systems and search functionalities. A detailed description of the search queries for each database is available in the supplementary file to ensure transparency and reproducibility.

### Study selection

Two independent researchers (TRCML and BCLA) selected the studies based on the title and abstract of each article using the Rayyan software for systematic reviews. Relevant studies were read in full and selected according to the eligibility criteria. Discrepancies between the two reviewers were resolved by consensus or, when necessary, by consulting a third reviewer (PRMF), an expert in systematic reviews and meta-analyses, to ensure methodological rigor in study selection.

### Data extraction

Two authors (TRCML and BCLA) independently extracted data from the included studies using a standardized form. Subsequently, the authors verified the accuracy of their respective forms. In cases of disagreement, the third reviewer (PRMF) adjudicated and resolved the discrepancies to ensure the accuracy of the extracted data. The standardized data extraction form included the following information: author; year of publication; country, study design and objective; sample size and characteristics; diagnostic criteria for rhinitis and hearing loss; type of hearing loss; auditory thresholds at frequencies of 250Hz, 500Hz, 1000Hz, 2000Hz, 4000Hz, and 8000Hz; latencies of waves I, III, and V from Brainstem Evoked Response Audiometry (BERA); and latencies of interpeak intervals I-III, III-V, and I-V. For each study, the mean and standard deviation (SD) of the outcomes of interest were extracted. In cases where the values of auditory thresholds and brainstem auditory evoked potentials were provided for each ear, a single mean and SD were calculated for the meta-analysis.

### Outcomes

The primary outcome of this study was the difference in the means of auditory thresholds at frequencies from 250Hz to 8000Hz measured by audiometry between patients with rhinitis compared to the control group. The secondary outcomes were the differences between the groups in terms of the means of the latencies of waves I, III, and V, and interpeak intervals I-III, III-V, and I-V.

### Risk of bias assessment

The Joanna Briggs Institute Critical Appraisal Checklist for Analytical Cross-Sectional Studies (https://jbi.global/critical-appraisal-tools) was used to identify potential biases in individual studies. This evaluation focused on various aspects, including eligibility criteria, auditory characteristics of the patients, use of diagnostic methods for rhinitis and hearing loss, potential confounding factors, and application of appropriate statistical analysis. The risk of bias assessment was also performed independently by two reviewers (BCLA and SMS), and discrepancies were resolved by consensus or, when necessary, through consultation with a third reviewer (PRMF) to guarantee consistency in the evaluation process.

### Meta-analysis

In this study, the meta-analysis was conducted using the inverse variance method under a random effects model, with statistical heterogeneity among the studies quantified by the I^2^ index^(16)^. Given the continuous nature and similarity with which the outcomes of interest were measured in individual studies, the weighted mean difference (MD) between the two comparison groups was used as the effect size. To assess the robustness of the findings, a leave-one-out sensitivity analysis was performed, sequentially removing each study from the meta-analysis to determine its influence on the overall estimates. Subgroup analyses were conducted to explore potential sources of heterogeneity. The meta-analysis was performed using RevMan software (version 5.4; Cochrane Collaboration).

### Level of available evidence

The level of evidence available in this meta-analysis was assessed using the GRADE system (Grading of Recommendations Assessment, Development and Evaluation)^(17)^. This system is recognized for providing a systematic and transparent approach to evaluating the quality of evidence and the strength of recommendations in health research. Through GRADE, evidence is classified into four levels—high, moderate, low, and very low—based on criteria such as the risk of bias, consistency among studies included in the evaluation of the outcomes of interest, the precision of the results, the directness of the evidence, the likelihood of publication bias, the effect size, the presence of a dose-response gradient, and the influence of confounding factors on the effect estimate. In this review, an analysis of a dose-response gradient and the influence of residual confounding factors were not conducted. Additionally, an assessment of potential publication bias was not carried out due to the limited number of studies available^(18)^.

## RESULTS

### Study selection

**Figure 1:**
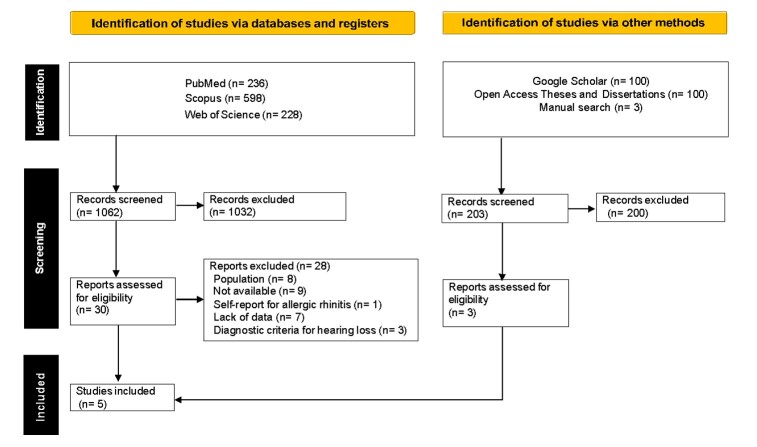
Flow chart for the literature review process

The initial search yielded 1,265 registers. After a thorough review of titles and abstracts, 30 articles were selected for full reading. Of these, two met the eligibility criteria^(19,20)^ and 28 were excluded for various reasons: eight did not involve patients with rhinitis; nine were not available in the databases, despite efforts to contact the authors; one was a self-report for hearing loss; seven did not provide essential information, such as audiometric thresholds or the number of patients with rhinitis and hearing loss; and three did not mention the diagnostic criteria for hearing loss or used questionnaires for this purpose (Supplementary file). Three studies were included following a manual search, which involved reviewing the reference lists of the selected articles^(10,21,22)^. Finally, five studies were included in this systematic review^(10,19-22)^ (Figure 1).

### Characteristics of the studies

Of the five included studies, three were conducted in India^(10,19,20)^ and two in Turkey^(21,22)^, published from 2011 to 2022. The total sample comprised 432 patients, 291 diagnosed with allergic rhinitis and 141 without rhinitis. The age distribution of the participants ranged from six to 58 years, encompassing both children and adults. The diagnosis of allergic rhinitis was clinically established in all the studies evaluated. Specific details and characteristics of the studies are systematized in Table 1.

**Table 1:** Characteristics of the studies included in the systematic review

**Author, Country**	**Objective**	**Population**	**Patients with rhinitis**	**Age range (mean ± SD)**	**Diagnosis of allergic rhinitis**	**Hearing assessment**	**Tested Frequencies**
Mahajan et al.^(20)^, India	To assess the audiological profile of patients with allergic rhinitis	Children and adults	100	10-55y	Clinical examination and medical history	Audiometry and BERA	250Hz to 8000Hz
				(32.9y ± 12.4)			
		(n = 150)					
Sekhon et al.^(10)^, India	To estimate the prevalence of audiological abnormalities in patients with upper airway allergy	Children and adults	53	12-58y	Clinical examination and medical history	Audiometry and BERA	250Hz to 8000Hz*
				(25.8y)			
		(n = 73)					
Nursoy et al.^(22)^, Turkey	To investigate the impact of allergy on hearing functions in children with allergic rhinitis	Children	50	6-15y	Clinical symptoms and skin prick tests	Audiometry and BERA	250Hz to 8000Hz*
		(n = 70)		(10.4y)			
Karabulut et al.^(21)^, Turkey	To investigate hearing function in patients with allergic rhinitis	Adults	58	18-40y	Clinical symptoms and skin prick tests	Audiometry	250Hz to 8000Hz*
		(n = 89)		(27.7y ± 6.0)			
Singh et al.^(19)^, India	To assess the otological and audiological status of patients with allergic rhinitis	Adults	30	17-45y	Clinical examination and medical history	Audiometry and BERA	250Hz to 8000Hz
		(n = 50)		(31y)			

* The 6000 Hz frequency has not been tested

**Caption:** BERA = Brainstem Evoked Response Audiometry; SD = standard deviation

### Risk of bias

The risk of bias assessment among the included studies revealed both strengths and issues that could compromise the validity and reliability of their results. Generally, the inclusion criteria were clearly defined, the subjects of the study and the setting were described in detail, and the outcomes (auditory thresholds and brainstem auditory evoked responses) were measured in a valid and reliable manner across all analyzed studies. These positive aspects are crucial as they ensure proper participant selection and accuracy in the measurements of audiological outcomes.

However, the analysis also identified significant methodological inconsistencies. The lack of explicit strategies for dealing with confounding factors was a recurring issue. Most studies did not mention the use of adjustment techniques, such as multivariate regression or stratification, limiting the ability to control external variables that could influence the results. Furthermore, many studies did not provide sufficient details about the standardized and recognized criteria for diagnosing allergic rhinitis consistently applied to the study and control groups, compromising the validity of the exposure measurements (Table 2) (Supplementary file).

**Table 2:** Risk of bias assessment

**Author**	**Q1**	**Q2**	**Q3**	**Q4**	**Q5**	**Q6**	**Q7**	**Q8**
Mahajan et al.^(20)^	●	●	●	●	●	●	●	●
Sekhon et al.^(10)^	●	●	●	●	●	●	●	●
Nursoy et al.^(22)^	●	●	●	●	●	●	●	●
Karabulut et al.^(21)^	●	●	●	●	●	●	●	●
Singh et al.^(19)^	●	●	●	●	●	●	●	●

● = Low risk of bias; ● = Unclear; ● = High risk of bias

**Caption:** Q1 = Were the criteria for inclusion in the sample clearly defined?; Q2 = Were the study subjects and the setting described in detail?; Q3 = Was the allergic rhinitis measured in a valid and reliable way?; Q4 = Were objective, standard criteria used for the diagnosis of allergic rhinitis applied consistently to both groups?; Q5 = Were confounding factors identified?; Q6 = Were strategies to deal with confounding factors stated?; Q7 = Were the outcomes (hearing thresholds and auditory brainstem responses) measured in a valid and reliable way?; Q8 = Was appropriate statistical analysis used?

### Meta-analysis

**Figure 2:**
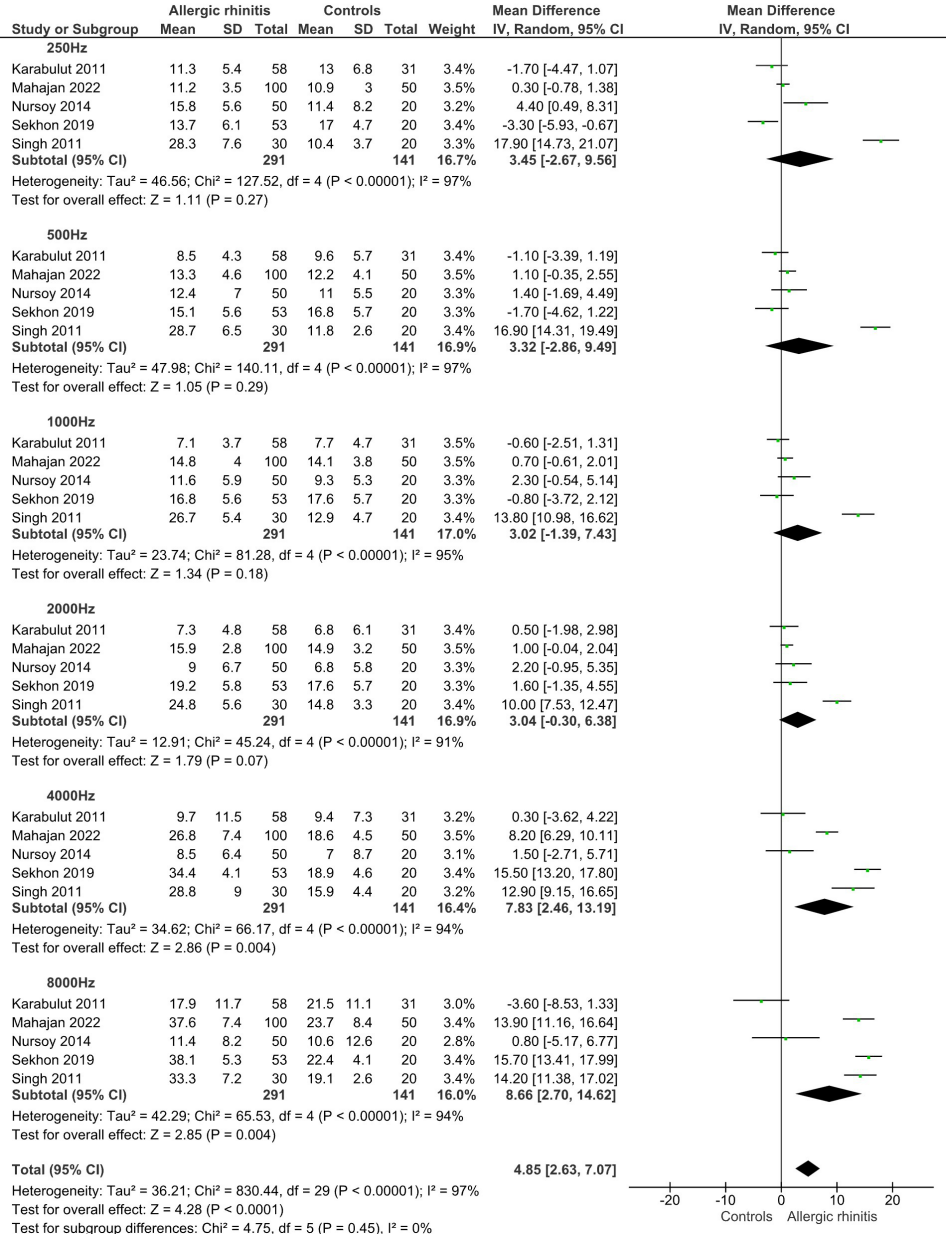
Meta-analysis summarizing the weighted mean differences between patients with and without allergic rhinitis in relation to audiometry results

The meta-analysis revealed that individuals with allergic rhinitis had significantly higher auditory thresholds compared to controls, with a pooled mean difference (MD) of 4.85 dB (95% CI: 2.63 to 7.07; p < 0.001). Substantial heterogeneity was observed across studies (I^2^ = 97%). To investigate potential sources of heterogeneity, a subgroup analysis was conducted based on auditory frequency. The analysis showed that the overall effect was primarily driven by significant differences at 4000Hz (MD = 7.83 dB; 95% CI: 2.46 to 13.19; p = 0.004) and 8000Hz (MD = 8.66 dB; 95% CI: 2.70 to 14.62; p = 0.004), while no significant differences were found at lower frequencies (250Hz to 2000Hz) (Figure 2).

**Figure 3:**
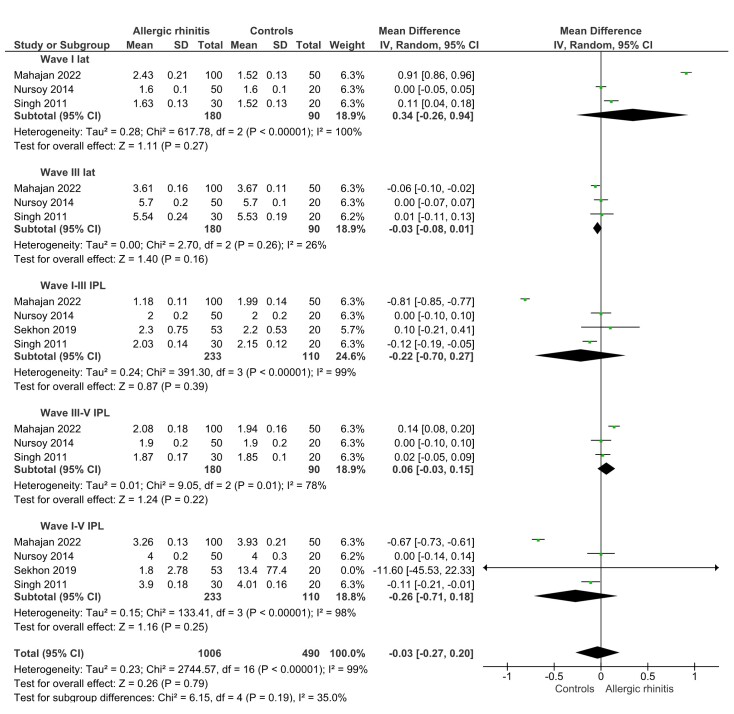
Meta-analysis summarizing the weighted mean differences between patients with and without allergic rhinitis in relation to auditory brainstem response potentials

Regarding BERA, the overall meta-analysis did not reveal a significant difference between groups (MD = –0.03 ms; 95% CI: –0.27 to 0.20; p = 0.79; I^2^ = 99%). Subgroup analysis by individual electrophysiological parameters confirmed the absence of significant differences for wave I (MD = 0.34 ms; 95% CI: –0.26 to 0.94; p = 0.27), wave III (MD = –0.03 ms; 95% CI: –0.08 to 0.01; p = 0.16), and interpeak intervals I–III, III–V, and I–V (Figure 3). In addition, only one study^(20)^ provided data on wave V latency, reporting statistically significant prolongation in the allergic rhinitis group (MD = 0.08 ms; 95% CI: 0.02 to 0.14; p = 0.01). However, given the reliance on a single study and the small magnitude of the effect, this finding must be interpreted with caution.

Caption: SD = standard deviation; CI = confidence interval; IV = inverse variance.

Subgroup analysis by region revealed that statistically significant differences in auditory thresholds were observed only in studies conducted in India, particularly at 4000 Hz (MD = 12.14 dB; 95% CI: 7.13 to 17.16; I^2^ = 92%) and 8000 Hz (MD = 14.75 dB; 95% CI: 13.25 to 16.24; I^2^ = 0%), while no significant differences were found in studies conducted in Turkey. Regarding BERA outcomes, regional subgroup analysis did not demonstrate significant differences in wave latencies or interpeak intervals in either country, reinforcing the absence of a consistent impact of allergic rhinitis on auditory brainstem processing (Supplementary file).

### Certainty of evidence

The quality of evidence was rated as “very low” regarding the differences in hearing thresholds at 4000Hz and 8000Hz frequencies between individuals with and without allergic rhinitis, as well as for the overall auditory threshold meta-analysis (Table 3).

**Table 3:** Strength of evidence for differences in overall hearing thresholds and at 4000Hz and 8000Hz frequencies between individuals with and without allergic rhinitis

**Frequency**	**Effect size (95% CI)**	**I^2^**	**Risk of bias**	**Inconsistency**	**Indirect evidence**	**Imprecision**	**Magnitude of effect**	**Strength of evidence**
Overall effect	MD 4.85	97%	Serious	Very serious	Non serious	Serious	Large	⨁◯◯◯
	(2.63 to 7.07)							Very low
4000Hz	MD 7.83	94%	Serious	Very serious	Non serious	Very serious	Very large	⨁◯◯◯
	(2.46 to 13.19)							Very low
8000Hz	MD 8.66	94%	Serious	Very serious	Non serious	Very serious	Very large	⨁◯◯◯
	(2.70 to 14.62)							Very low

Strength of evidence: ⨁ very low; ⨁⨁ low; ⨁⨁⨁ moderate; ⨁⨁⨁⨁ high

**Caption:** CI = Confidence interval; MD = Mean Difference

## DISCUSSION

The aim of this systematic review was to investigate potential differences in auditory thresholds and brainstem auditory evoked responses between individuals with and without allergic rhinitis, providing evidence to support clinical decision-making. The meta-analysis revealed that individuals with allergic rhinitis had higher auditory thresholds, particularly at 4000Hz and 8000Hz, while no consistent differences were found in BERA parameters. These results suggest that allergic rhinitis may affect peripheral auditory function, especially at higher frequencies, without significantly impairing neural conduction at the brainstem level.

Subgroup analysis by geographic location revealed that significant auditory threshold differences were observed only in studies conducted in India, whereas studies from Turkey did not show statistically significant results. These regional discrepancies may be attributable to environmental exposures (e.g., air pollution, allergen profiles)^(23)^ or genetic predisposition^(24-26)^. Furthermore, clinical and methodological factors—such as participant age range, clinical severity of allergic rhinitis, and audiological protocols—may also contribute to these divergent findings. The presence of significant effects only at higher frequencies suggests a localized cochlear involvement, possibly affecting outer hair cells or cochlear microcirculation^(19)^.

The high heterogeneity observed in the BERA outcomes warrants cautious interpretation. Differences in BERA findings across studies likely stem from methodological inconsistencies, including variability in equipment, stimulus parameters, recording protocols, and criteria for diagnosing allergic rhinitis. These factors may explain the inconsistent direction and magnitude of the effect sizes across studies. However, the lack of consistent abnormalities in wave latencies or interpeak intervals suggest that allergic rhinitis may not significantly affect auditory brainstem conduction.

Several mechanisms may explain the association between allergic rhinitis and elevated auditory thresholds. Allergic inflammation can lead to Eustachian tube dysfunction due to mucosal edema or obstruction, resulting in negative middle ear pressure and accumulation of secretions^(27,28)^, which may predispose to otitis media with effusion (OME). This condition is particularly prevalent in pediatric populations with allergic rhinitis and may contribute to conductive hearing loss^(28-30)^. Additionally, the detection of allergen-specific IgE in middle ear effusions supports a local immunological mechanism contributing to otological alterations^(31)^.

Beyond conductive mechanisms, cochlear involvement may also occur. Chronic allergic inflammation can induce vascular changes affecting the stria vascularis and alter the ionic composition of endolymph, which is critical for high-frequency hearing^(32)^. Local immune responses in the inner ear, including direct cochlear inflammation or neuroinflammatory activity (e.g., microglial activation)^(12,33)^​, may further compromise outer hair cell function. These mechanisms are compatible with the selective impairment observed at higher frequencies.

Despite these findings, the overall quality of evidence was rated as very low, mainly due to methodological limitations, high heterogeneity, and imprecision. These limitations reduce the generalizability of the results. The wide confidence intervals and variability in diagnostic methods highlight the need for standardized protocols in future research. Nonetheless, the observed magnitude of the effect at higher frequencies suggests potential clinical relevance.

## CONCLUSION

This systematic review and meta-analysis identified higher auditory thresholds at 4000Hz and 8000Hz in individuals with allergic rhinitis, suggesting a potential peripheral auditory effect. No consistent differences were observed in BERA parameters, indicating that allergic rhinitis may not affect neural conduction at the brainstem level. Further high-quality longitudinal studies are necessary to validate these associations and elucidate underlying mechanisms.

## Supplementary Material

Supplementary material accompanies this paper.

Supplementary File Auditory function and brainstem responses in allergic rhinitis: systematic review and meta-analysis.

This material is available as part of the online article from https://doi.org/10.1590/2317-1782/e20240307en
