# Supplementary material for: Auditory function and brainstem responses in allergic rhinitis: systematic review and meta-analysis
Source: Codas. 2025 Oct 13;37(5):e20240307. doi: 10.1590/2317-1782/e20240307en (PMC12533529; doi:10.1590/2317-1782/e20240307en)
Supplement: Supplementary File [file codas-37-5-e20240307-suppl01.pdf]

SUPPLEMENTARY FILE

AUDITORY FUNCTION AND BRAINSTEM RESPONSES IN ALLERGIC RHINITIS: SYSTEMATIC REVIEW AND META-ANALYSIS

PRISMA 2020 Checklist: Completeness of Items

TITLE

Does the title identify the article as a systematic review?

The title clearly identifies the study as a systematic review and meta-analysis.

ABSTRACT

Does the abstract adhere to the PRISMA checklist for abstracts?

The abstract complies with the PRISMA guidelines.

INTRODUCTION

Rationale: Does it justify the review in the context of existing knowledge?

The introduction provides a strong justification, citing prior evidence and gaps in the literature.

Objectives: Are the objectives of the review explicitly stated?

The objectives are clearly and explicitly stated.

METHODS

Eligibility Criteria: Does it specify inclusion and exclusion criteria?

Inclusion and exclusion criteria are well-defined, including study type, population, comparators, and outcomes.

Information Sources: Are all sources of data specified?

The manuscript specifies databases and grey literature sources, including search dates.

Search Strategy: Is the full search strategy presented?

The full search strategy is described in the text.

Selection Process: Are the methods for determining study inclusion detailed?

The manuscript describes dual independent review and resolution of discrepancies by a third reviewer.

Data Collection Process: Are the methods for data extraction explained?

The process of independent data extraction and verification by two reviewers is described.

Data Items: Are all sought outcomes and variables listed and defined?

The manuscript lists all collected variables, including population characteristics and audiometric measures.

Study Risk of Bias Assessment: Are methods for risk of bias assessment specified?

The JBI tool is described in detail.

Effect Measures: Are the measures of effect for each outcome detailed?

The weighted mean difference (MD) is specified as the measure of effect.

Synthesis Methods: Are methods for synthesis and heterogeneity described?

Methods for meta-analysis are described, including subgroup analysis, sensitivity analyses, and exploration of heterogeneity.

Reporting Bias Assessment: Are methods for assessing reporting bias described?

Due to the limited number of studies, a formal assessment of reporting bias was not performed.

Certainty Assessment: Are methods for assessing certainty of evidence described?

The GRADE system is used for evaluating evidence quality.

RESULTS

Study Selection: Are the results of the selection process described?

A flow diagram and details of included/excluded studies are provided.

Study Characteristics: Are the characteristics of included studies presented?

Table 1 provides detailed information about the included studies.

Risk of Bias in Studies: Is the risk of bias assessment presented?

Table 2 outlines the risk of bias for each study.

Results of Individual Studies: Are summary statistics for each study presented?

The results of individual studies are described in the forest plots.

Results of Syntheses: Are synthesis results clearly presented?

The results of the meta-analysis are clearly described in the text and forest plots, including a subgroup analysis.

Reporting Biases: Are assessments of reporting bias presented?

No.

Certainty of Evidence: Are certainty assessments presented?

Certainty assessments are presented in the text and Table 3.

DISCUSSION

Discussion: Are results interpreted in the context of other evidence?

The discussion integrates findings with prior evidence and highlights limitations.

Implications: Are implications for practice, policy, and research discussed?

Implications are clearly stated for clinical practice and future research.

OTHER INFORMATION

Registration and Protocol: Are registration and protocol details provided?

The protocol of this systematic review and meta-analysis was registered on the Open Science Framework.

Support: Are sources of support described?

Funding and conflicts of interest are disclosed.

Availability of Data, Code, and Other Materials: Are materials publicly available?

Data are available on reasonable request.

Search Strategy

DATABASE	STRATEGY
**PubMed**	(Rhinitis[Mesh] OR Rhinitis[All Fields] OR Rhinitides[All Fields]) AND ("Hearing Loss"[Mesh] OR "Hearing Loss"[All Fields] OR "Hearing Disorders"[Mesh] OR "Hearing Disorders"[All Fields] OR "Hearing Disorder"[All Fields] OR "Hearing Impairment"[All Fields] OR Deafness[Mesh] OR Deafness[All Fields] OR Hypoacusis[All Fields] OR Hypoacuses[All Fields] OR "Deaf Mutism"[All Fields] OR "Deaf-Mutism"[All Fields])
**Scopus**	(Rhinitis OR Rhinitides) AND ("Hearing Loss" OR "Hearing Disorders" OR "Hearing Disorder" OR "Hearing Impairment" OR Deafness OR Hypoacusis OR Hypoacuses OR "Deaf Mutism" OR "Deaf-Mutism")
**Web of Science**	(Rhinitis OR Rhinitides) AND ("Hearing Loss" OR "Hearing Disorders" OR "Hearing Disorder" OR "Hearing Impairment" OR Deafness OR Hypoacusis OR Hypoacuses OR "Deaf Mutism" OR "Deaf-Mutism")
**Google Scholar**	(Rhinitis OR Rhinitides) AND ("Hearing Loss" OR "Hearing Disorders" OR "Hearing Disorder" OR "Hearing Impairment" OR Deafness OR Hypoacusis OR Hypoacuses OR "Deaf Mutism" OR "Deaf-Mutism")
**Open Access Theses and Dissertations (OATD)**	(Rhinitis OR Rhinitides) AND ("Hearing Loss" OR "Hearing Disorders" OR "Hearing Disorder" OR "Hearing Impairment" OR Deafness OR Hypoacusis OR Hypoacuses OR "Deaf Mutism" OR "Deaf-Mutism")

Excluded Studies and Reasons

Author	Year	Title	Journal	DOI	Reason for exclusion
Prabakaran	2024	Audiological Profile in Allergic Rhinitis, a Hospital Based Study	Indian Journal of Otolaryngology and Head & Neck Surgery	10.1007/s12070-023-04066-9	The hearing threshold values could not be extracted
Navin	2024	Ent Manifestations in Sculptors of South Chennai, India: A Cross-Sectional Observational Study	Indian Journal of Otolaryngology and Head & Neck Surgery	10.1007/s12070-023-04179-1	It was not possible to extract the number of individuals with hearing loss among those with rhinitis
Kuan	2023	Identifying and visualising multimorbidity and comorbidity patterns in patients in the English National Health Service: a population-based study	Lancet Digital Health	10.1016/S2589-7500(22)00187-X	The diagnostic criterion for hearing loss was not mentioned
Sahni	2022	Hearing Assessment in Patients of Allergic Rhinitis: A Study on 200 Subjects	Indian Journal of Otolaryngology and Head & Neck Surgery	10.1007/s12070-020-01890-1	The hearing threshold values could not be extracted
Choi	2022	Association of sudden sensorineural hearing loss with asthma: a longitudinal follow-up study using a national sample cohort	BMJ Open	10.1136/bmjopen-2020-047966	The study included individuals diagnosed with asthma
Xie	2020	Estimated Prevalence of Asthma in US Children with Developmental Disabilities	JAMA Network Open	10.1001/jamanetworkopen.2020.7728	The study included individuals diagnosed with asthma
Dwarakanath	2019	Assessment of Hearing in Individuals with Allergic Rhinitis	Indian Journal of Otology	10.4103/indianjotol.INDIANJOTOL_12_19	The hearing threshold values could not be extracted
McKee	2018	Hearing loss and associated medical conditions among individuals 65 years and older	Disability and Health Journal	10.1016/j.dhjo.2017.05.007	The study included individuals diagnosed with asthma
Arif	2016	The association of childhood asthma with mental health and developmental comorbidities in low-income families	Journal of Asthma	10.3109/02770903.2015.1089277	The study included individuals diagnosed with asthma
Kilic	2014	Evaluation of auditory functions in patients with asthma	European Review for Medical and Pharmacological Sciences	-	The study included individuals diagnosed with asthma
James	2013	Obesity and asthma at school entry: Co-morbidities and temporal trends	Journal of Paediatrics and Child Health	10.1111/jpc.12160	The study included individuals diagnosed with asthma
Arif	2010	The Association Between Symptomatic Asthma and Neurobehavioral Comorbidities Among Children	Journal of Asthma	10.3109/02770903.2010.491148	The study included individuals diagnosed with asthma
Sultész	2010	Prevalence and risk factors for allergic rhinitis in primary schoolchildren in Budapest	International Journal of Pediatric Otorhinolaryngology	10.1016/j.ijporl.2010.02.008	Self-report for hearing loss
Oghan	2008	Does the prevalence of otolaryngological diseases in deaf children differ from children without hearing impairment?	European Archives of Oto-Rhino-Laryngology	10.1007/s00405-007-0426-6	The diagnostic criterion for hearing loss was not mentioned

Steinsvåg	2007	Nasal symptoms and signs in children suffering from asthma	International Journal of Pediatric Otorhinolaryngology	10.1016/j.ijporl.2006.12.012	The study included individuals diagnosed with asthma
Rózańska-Kudelska	2005	Assessment of the hearing organ in the patients with allergic perennial and seasonal allergic rhinitis	Polish Journal of Otolaryngology	-	Full text not provided
Sobki	2004	Point prevalence of allergic rhinitis among Saudi children	Rhinology	-	WHO questionnaire for hearing loss
Modrzyński	2003	The results of tympanometry in children with adenoid hypertrophy and coexisting allergy	Przeglad Lekarski	-	Full text not provided
Matusiak	2002	Prevalence of conductive hypoacusis in children aged 5-9 years old from rural area in Poland--prospective screening of healthy subjects	Polish Journal of Otolaryngology	-	Full text not provided
Chopra	1999	Deafness in Paediatric Age Group	Indian Journal of Otolaryngology and Head & Neck Surgery	10.1007/BF02996520	It was not possible to extract the number of individuals with hearing loss among those with rhinitis
Zakzouk	1999	A survey of childhood hearing impairment	Saudi Medical Journal	-	Full text not provided
Walker	1998	Allergic rhinitis history as a predictor of other future disqualifying otorhinolaryngological defects	Aviation, Space, and Environmental Medicine	-	Full text not provided
Ruggeri	1990	An assessment of middle ear involvement in children with allergic rhinitis: a comparison with chronic hypertrophic adenoiditis	La Pediatria Medica e Chirurgica	-	Full text not provided
Sorri	1986	Respiratory tract disease and hearing loss. Preliminary report	Scandinavian Audiology	-	Full text not provided
Baker	1982	Vocal Quality, Articulation and Audiological Characteristics of Children and Young Adults with Diagnosed Allergies	Annals of Otology, Rhinology & Laryngology	10.1177/0003489482091003	The number of patients with rhinitis was not provided
Uetsuka	1970	Rhinitis and Hearing Loss in Schoolchildren	Oto-Rhino-Laryngology Tokyo	10.11453/orltokyo1958.13.627	Full text not provided
Szanton	1966	Hearing disturbances in allergic children	Journal of Asthma Research	10.3109/02770906609100303.	It was not possible to extract the number of individuals with hearing loss among those with rhinitis
Clerici	1953	Behavior of the auditory function during allergic rhinopathy	Archivio Italiano di Otologia, Rinologia e Laringologia	-	Full text not provided

Risk of Bias Assessment

Mahajan A, Manhas M, Kalsotra P, Kalsotra G, Gul N. A prospective study of audiological manifestations in patients of allergic rhinitis. Indian J Otolaryngol Head Neck Surg. 2022 Oct;74(Suppl 2): S1256–S1261;. DOI: 10.1007/s12070-020-02343-5.

1. Were the criteria for inclusion in the sample clearly defined?

Yes. The inclusion criteria are clearly defined in the study. Patients aged between 10 and 55 years with a clinical diagnosis of allergic rhinitis, based on signs and symptoms, were included. The comparison group consisted of individuals of similar age, exposed to the same environment, but without allergic rhinitis or systemic diseases.

2. Were the study subjects and the setting described in detail?

Yes. The study subjects and setting were described in detail. The study was conducted in the Department of Otolaryngology at SMGS Hospital, Government Medical College, Jammu. Demographic characteristics of the participants, such as age and gender distribution, were provided.

3. Was the allergic rhinitis measured in a valid and reliable way?

Unclear. The diagnosis of allergic rhinitis was based on a complete clinical examination and the medical history of the patients, but the study does not specify whether validated methods, such as skin tests or specific IgE measurements, were used to confirm the exposure.

4. Were objective, standard criteria used for the diagnosis of allergic rhinitis applied consistently to both groups?

Unclear. Although the diagnosis was based on clinical examination and medical history, there are not enough details about the use of standardized and recognized criteria applied consistently to both groups (allergic rhinitis group and control group).

5. Were confounding factors identified?

No. Although the study excluded participants with some potential confounding factors, it does not comprehensively discuss all possible confounding factors and how these could be identified and adjusted for in the data analysis. Simply excluding participants does not fully address the identification and control of confounding factors.

6. Were strategies to deal with confounding factors stated?

No. The study does not mention specific strategies to deal with confounding factors in the data analysis, such as the use of adjusted analyses or stratification techniques to control for these factors. The analyses seem to focus mainly on direct comparisons between the study and control groups.

7. Were the outcomes (hearing thresholds and auditory brainstem responses) measured in a valid and reliable way?

Yes. The article details the methods of auditory measurement, indicating the use of standardized equipment and specific procedures to ensure the accuracy and reliability of the measurements.

8. Was appropriate statistical analysis used?

Unclear. The absence of details on the specific statistical tests used to analyze the data prevents a full assessment of the adequacy of the statistical analyses. The lack of specification of statistical methods compromises the transparency and replicability of the results.

Sekhon GS, Verma R, Munjal M, Chopra H. Audiological manifestations in patients of upper airway allergy. Int J Otorhinolaryngol Head Neck Surg. 2019 Nov-Dec;5(6):1451-1456. DOI: 10.18203/issn.2454-5929.ijohns20194542.

1. Were the criteria for inclusion in the sample clearly defined?

Yes. The inclusion and exclusion criteria were clearly defined in the article. Patients diagnosed with upper airway allergies were selected based on ear, nose, throat, and systemic examinations, as well as clinical history.

2. Were the study subjects and the setting described in detail?

Yes. The study sample was described in detail, including the demographics of the participants, location (Dayanand Medical College and Hospital, Ludhiana, Punjab, India), and the study period (January 2012 to December 2012). The characteristics of the study subjects were provided, including average age and gender distribution.

3. Was the allergic rhinitis measured in a valid and reliable way?

Unclear. The article mentions that the diagnosis of allergic rhinitis was based on a complete clinical examination and the medical history of the patients, but it does not specify whether validated criteria or a standard method were used to confirm the diagnosis.

4. Were objective, standard criteria used for the diagnosis of allergic rhinitis applied consistently to both groups?

Unclear. Similar to question 3, the article mentions that the diagnosis was based on clinical examination and medical history, but it does not specify whether standardized and recognized criteria were used, applied consistently to both groups.

5. Were confounding factors identified?

No. Although the study excluded participants with some potential confounding factors, it does not comprehensively discuss all possible confounding factors and how they could be identified and adjusted for in the data analysis. Simply excluding participants does not fully address the identification and control of confounding factors.

6. Were strategies to deal with confounding factors stated?

No. Specific strategies to deal with confounding factors in the data analysis were not described. There is no mention of adjustment techniques such as multivariate regression or stratification to control the effects of potential confounders. The statistical analyses mentioned focus primarily on direct comparisons between the study and control groups, without explicit adjustment for confounders.

7. Were the outcomes (hearing thresholds and auditory brainstem responses) measured in a valid and reliable way?

Yes. The article details the auditory measurement methods, indicating the use of standardized equipment and specific procedures to ensure the accuracy and reliability of the measurements.

8. Was appropriate statistical analysis used?

Yes. Statistical analysis was performed using appropriate tests for group comparisons. Student's t-tests were used for normally distributed data and the Mann-Whitney test for skewed data. p-values were considered significant if less than 0.05.

Nursoy MA, Aksoy F, Dogan R, Ozturan O, Eren SB, Veyseller B, Ozkaya E, Demir AD, Ozturk B. Audiological findings in pediatric perineal allergic rhinitis (house dust mite allergy) patients. Eur Arch Otorhinolaryngol. 2013;270(4):1369-1374. DOI: 10.1007/s00405-013-2628-4.

1. Were the criteria for inclusion in the sample clearly defined?

Yes. The inclusion criteria were clearly defined. The study included 50 pediatric patients diagnosed with perennial allergic rhinitis, aged between 6 and 15 years, and a control group of 20 healthy individuals in the same age range. The exclusion criteria included a history of noise exposure, use of ototoxic medications, metabolic, neurological, or other otolaryngological pathologies.

2. Were the study subjects and the setting described in detail?

Yes. The study subjects and the setting were described in detail. Patients were recruited from the outpatient pediatric clinic of Bezmialem Vakif University, and the study included demographic details of the participants, such as age and gender distribution.

3. Was the allergic rhinitis measured in a valid and reliable way?

Yes. Allergic rhinitis was diagnosed based on clinical symptoms and skin prick tests.

4. Were objective, standard criteria used for the diagnosis of allergic rhinitis applied consistently to both groups?

Yes. The objective and standardized criteria for the diagnosis of allergic rhinitis (skin tests) were applied consistently to the study group and the control group to confirm the absence of allergy in the control group and the presence in the study group.

5. Were confounding factors identified?

No. Although the study excluded participants with some potential confounding factors, it does not comprehensively discuss all possible confounding factors and how they could be identified and adjusted for in the data analysis. Simply excluding participants does not fully address the identification and control of confounding factors.

6. Were strategies to deal with confounding factors stated?

No. Specific strategies to deal with confounding factors in the data analysis were not described. There is no mention of adjustment techniques such as multivariate regression or stratification to control the effects of potential confounders. The statistical analyses mentioned focus primarily on direct comparisons between the study and control groups, without explicit adjustment for confounders.

7. Were the outcomes (hearing thresholds and auditory brainstem responses) measured in a valid and reliable way?

Yes. The outcomes were measured in a valid and reliable way. Standardized audiological methods were used, in compliance with the standards of the American National Standards Institute (ANSI) and the International Organization for Standardization (ISO).

8. Was appropriate statistical analysis used?

Yes. Independent samples t-tests were used to compare means between the groups, and data normality was verified using the Kolmogorov-Smirnov tests. A p-value of less than 0.05 was considered significant.

Karabulut H, Acar B, Dagli M, Karadag AS, Baysal S, Karasen RM. Investigation of hearing in patients with allergic rhinitis. Iran J Allergy Asthma Immunol. 2011 Mar;10(1):29-33. DOI: 10.14735/amko2017en7.

1. Were the criteria for inclusion in the sample clearly defined?

Yes. The inclusion and exclusion criteria were clearly specified, covering the confirmation of allergic rhinitis through a positive skin test and the exclusion of factors that could influence auditory results.

2. Were the study subjects and the setting described in detail?

Yes. The study subjects and the setting were described in detail. Patients were recruited from the Kecioren Training and Research Hospital in Ankara, Turkey. Demographic information of the participants, such as age and gender distribution, was provided.

3. Was the allergic rhinitis measured in a valid and reliable way?

Yes. Allergic rhinitis was diagnosed based on clinical symptoms and standardized skin test results, which are valid and reliable methods for confirming allergen exposure.

4. Were objective, standard criteria used for the diagnosis of allergic rhinitis applied consistently to both groups?

Yes. The objective and standardized criteria for the diagnosis of allergic rhinitis (skin tests) were applied consistently to the study group and the comparison group.

5. Were confounding factors identified?

No. Although the study excluded participants with some potential confounding factors, it does not comprehensively discuss all possible confounding factors and how they could be identified and adjusted for in the data analysis. Simply excluding participants does not fully address the identification and control of confounding factors.

6. Were strategies to deal with confounding factors stated?

No. Although the study excluded participants with potential confounding factors, specific strategies to deal with confounding factors in the data analysis were not mentioned, such as the use of adjusted analyses or stratification techniques to control for these factors.

7. Were the outcomes (hearing thresholds and auditory brainstem responses) measured in a valid and reliable way?

Yes. The outcomes were measured in a valid and reliable way. Standardized audiological methods, such as pure tone audiometry and tympanometry, were used with appropriate equipment and standardized procedures to ensure the accuracy and reliability of the measurements.

8. Was appropriate statistical analysis used?

Yes. Mann-Whitney U tests were used for group comparisons and independent samples t-tests were used to compare the ages of patients and controls. The chi-square test was used to compare the amount of hearing loss and the gender of patients and controls. A p-value of less than 0.05 was considered significant.

Singh S, Nagarkar AN, Bansal S, Vir D, Gupta AK. Audiological manifestations of allergic rhinitis. J Laryngol Otol. 2011 Jun;125(9):906-910. DOI: 10.1017/S0022215111001137.

1. Were the criteria for inclusion in the sample clearly defined?

Yes. The inclusion criteria were clearly defined. The study included 30 patients with a diagnosis of allergic rhinitis, confirmed by detailed clinical history and ENT examination, and 20 healthy individuals in the control group. Patients with a history of noise exposure, use of ototoxic medications, metabolic problems, neurological issues, or other ENT conditions were excluded from the study.

2. Were the study subjects and the setting described in detail?

Yes. The study subjects and the setting were described in detail. Patients were recruited from the outpatient otolaryngology department of the Post Graduate Institute of Medical Education and Research in Chandigarh, India. Demographic characteristics of the participants, such as age and gender distribution, were provided.

3. Was the allergic rhinitis measured in a valid and reliable way?

Unclear. The diagnosis of allergic rhinitis was based on a complete clinical examination and the medical history of the patients, but the study does not specify whether validated methods, such as skin tests or specific IgE measurements, were used to confirm the exposure.

4. Were objective, standard criteria used for the diagnosis of allergic rhinitis applied consistently to both groups?

Unclear. Although the diagnosis was based on clinical examination and medical history, there are not enough details about the use of standardized and recognized criteria applied consistently to both groups (allergic rhinitis group and control group).

Auditory Thresholds	250Hz	500Hz	1000Hz	2000Hz
**Overall effect**	3.45 (-2.67, 9.56) (I^2=97%)	3.32 (-2.86, 9.49) (I^2=97%)	3.02 (-1.39, 7.43) (I^2=95%)	3.04 (-0.30, 6.38) (I^2=91%)
**India**	4.89 (-5.07, 14.86) (I^2=98%)	5.43 (-4.95, 15.80) (I^2=98%)	4.53 (-3.54, 12.60) (I^2=97%)	4.16 (-1.48, 9.80) (I^2=95%)
**Turkey**	1.19 (-4.78, 7.16) (I^2=84%)	-0.08 (-2.49, 2.33) (I^2=38%)	0.65 (-2.16, 3.47) (I^2=64%)	1.15 (-0.80, 3.10) (I^2=0%)
**Test for subgroup differences**	p = 0.53	p = 0.31	p = 0.37	p = 0.32
**BERA**	**Wave I lat**	**Wave III lat**	**Wave V lat**	**Wave I-III IPL**
**Overall effect**	0.34 (-0.26, 0.94) (I^2=100%)	-0.03 (-0.08, 0.01) (I^2=26%)	0.08 (0.02, 0.14) (I^2=NA)	-0.22 (-0.70, 0.27) (I^2=99%)
**India**	0.51 (-0.27, 1.29) (I^2=100%)	-0.05 (-0.10, 0.00) (I^2=14%)	0.08 (0.02, 0.14) (I^2=NA)	-0.29 (-0.85, 0.28) (I^2=99%)
**Turkey**	0.00 (-0.05, 0.05) (I^2=NA)	0.00 (-0.07, 0.07) (I^2=NA)	-	0.00 (-0.10, 0.10) (I^2=NA)
**Test for subgroup differences**	p = 0.20	p = 0.28	-	p = 0.33

5. Were confounding factors identified?

No. Although the study excluded participants with some potential confounding factors, it does not comprehensively discuss all possible confounding factors and how they could be identified and adjusted for in the data analysis. Simply excluding participants does not fully address the identification and control of confounding factors.

6. Were strategies to deal with confounding factors stated?

No. Although the study excluded participants with potential confounding factors, specific strategies to deal with confounding factors in the data analysis were not mentioned, such as the use of adjusted analyses or stratification techniques to control for these factors.

7. Were the outcomes (hearing thresholds and auditory brainstem responses) measured in a valid and reliable way?

Yes. The outcomes were measured in a valid and reliable way. Standardized audiological methods, such as pure tone audiometry, otoacoustic emissions, and auditory brainstem responses, were used with appropriate equipment and standardized procedures to ensure the accuracy and reliability of the measurements.

8. Was appropriate statistical analysis used?

Yes. Student's t-tests were used to compare means between the groups, and the Mann-Whitney test was applied for non-parametric data. A p-value of less than 0.05 was considered significant.

Subgroup Analysis

Subgroup analysis of differences in auditory thresholds and brainstem evoked response audiometry (MD, 95% CI) between allergic rhinitis and control groups.

BERA, Brainstem Evoked Response Audiometry. NA, Not Applicable.
